# The Role of Physiotherapy in Pediatric Palliative Care: A Systematic Review

**DOI:** 10.3390/children8111043

**Published:** 2021-11-12

**Authors:** Silvia Ortiz-Campoy, Cristina Lirio-Romero, Helena Romay-Barrero, David Martín-Caro Álvarez, Purificación López-Muñoz, Rocío Palomo-Carrión

**Affiliations:** 1 Physiotherapy Center in Pediatrics, 45003 Toledo, Spain; sortizc_31@outlook.es; 2 Department of Nursing, Physiotherapy and Occupational Therapy, Faculty of Physiotherapy and Nursing, University of Castilla-La Mancha, 45071 Toledo, Spain; Helena.Romay@uclm.es (H.R.-B.); David.MartinCaro@uclm.es (D.M.-C.Á.); Purificacion.Lopez@uclm.es (P.L.-M.); Rocio.Palomo@uclm.es (R.P.-C.); 3 Group of Researching in Physiotherapy in Toledo, GIFTO, 45071 Toledo, Spain; 4 Pediatric-Unit, Hemi-Child-Research (GIFTO), UCLM, 45071 Toledo, Spain

**Keywords:** child interaction, family, health care, palliative care, physical therapy

## Abstract

Pediatric palliative care (PPC) is a set of actions aimed at children who suffer from a severe or life-threatening disease to alleviate the symptoms of the disease and improve the quality of life of both the child and his/her family. One of the tools used to control symptoms is physiotherapy; however, its application in the child population has not been thoroughly studied. The main objective of this study was to gather, analyze, and critically evaluate the available scientific evidence on physiotherapy in children who require palliative care through a systematic review of the studies published in the last 10 years in the following databases: PubMed, Cochrane Library, PEDro, CINAHL, and Scopus. Of a total of 622 studies, the inclusion criteria were only met by seven articles, which were focused on the relationship between physiotherapy and PPC. This study analyzed: (1) the main pathologies treated, with a predominance of cerebral palsy and cancer; (2) the interventions applied, such as respiratory physiotherapy, neurological physiotherapy, therapeutic massage, and virtual reality; (3) the effects achieved in the child and his/her family, highlighting the control of symptoms and the improvement of the quality of life; and (4) the knowledge of the physiotherapists on PPC, observing that most of the professionals had not received training in this scope. The findings of this review indicate a lack of an adequate evidence foundation for physiotherapy in PPC.

## 1. Introduction

The World Health Organization (WHO) defines pediatric palliative care (PPC) as the actions aimed at those children who suffer from severe, chronic, progressive, disabling, advanced or life-threatening diseases, with the aim of reducing the suffering and improving the quality of life throughout the entire process, regardless of the state of the disease. These are active and integral cares focused on the prevention and alleviation of pain and other physical symptoms, which also provide the necessary support to the psychological, social, and spiritual aspects of the patient and his/her family [1].

The described models of PPC have been modified along the years, although, in the years 1998 and 2000, the WHO and the American Academy of Pediatrics, respectively, proposed a similar model in which they proposed that PPC should be initiated regardless of whether the child received treatment with curative purposes or not. Therefore, PPC are not exclusively aimed at children in terminal stage, as they are initiated when a life-threatening disease is diagnosed, and they are maintained throughout the entire evolution of the disease, even in the process of family grief if necessary [2,3,4]. Recognizing the inflection point, that is, the moment that marks the entry of the child into the decline phase within the trajectory of his/her disease, is important for adapting the therapeutic objectives, and this is where palliative treatment is most relevant [4]. Although PPC is strongly related to palliative assistance for adults, children require specialized palliative attention with a multidisciplinary approach, mainly due to the variability in the age of the patients, the fact that children are in continuous physical, emotional, and cognitive development, and the important role of the family [1].

The Association for Children with Life-threatening or Terminal Conditions and their Families (ACT) and the UK Royal College of Paediatrics and Child Health (RCPCH) created a guide for the development of PPC services, which classifies the diseases of children susceptible to receive palliative treatment into four groups (Table 1) [5].

Approximately 21 million children (including newborns, infants, children, and adolescents of up to 19 years of age), worldwide and annually, could require a palliative approach [6]. The prevalence rate estimated for children between 0 and 19 years of age who may need PPC due to a life-limiting or life-threatening disease is 10–16 cases per 10,000 children [5]. Applying this estimation to the Spanish population aged 0–19 years (8,837,102) [7], between 8837 and 14,139 children could suffer from a life-limiting disease and require palliative care. Of all the children who require PPC in Spain, around 2500–3000 die every year (33% due to cancer and 66% due to other life-limiting diseases, predominantly neurodegenerative, metabolic, and genetic diseases). Although childhood mortality has decreased in the last years, the prevalence of incurable diseases and disability has increased; thus, an increasing number of children survive in a situation of high vulnerability and fragility, in some cases for years [8].

However, only 15% of children could be receiving PPC, due to the fact that equity has not been reached yet in all the healthcare services of Spain, together with the social impact of a child’s death [6]. Most autonomous communities do not have any exclusive protocol for the pediatric population. When there is a case, the patient is usually attended to by the reference hospital service (hematology, oncology, neurology, etc.), the palliative care teams, and the primary care pediatrician. Only five autonomous communities contemplate specific pediatric palliative care in their range of services: Balearic Islands, Canary Islands, Catalonia, Valencian Community, and the Community of Madrid [8]. In the last few years, the resources specialized in PPC have increased, with the formation of new units and the expansion of the existing ones. Specifically in Castile La Mancha, in November 2020, a resolution was passed on PPC with the aim of creating a specific unit; moreover, there is an item from the 2021 budget assigned to it, although it has not been applied yet [9,10].

Furthermore, the control of symptoms is one of the most important and most care-demanding factors in PPC [2], including mainly musculoskeletal, respiratory, gastrointestinal, genitourinary, neurological, and psychological symptoms [11]. The European Association of Palliative Care (EAPC) highlights the importance of controlling the symptoms and achieving the maximum growth and development potential of the child through continuous physical rehabilitation; thus, every child who requires palliative care should have access to it [3,12]. Physiotherapy is one of the disciplines in charge of carrying out such physical rehabilitation, acting in a preventive, curative, or palliative manner depending on the progression of the disease and adapting the goals to improve the functionality, autonomy, and quality of life of the patient [13,14,15]. Therefore, physiotherapy is a valuable tool for controlling symptoms in PPC.

Thus, the present review was carried out with the main aim of gathering, analyzing, and critically evaluating the available scientific evidence on physiotherapy in children who require palliative care. A set of more specific objectives were set, in order to (1) determine the pathologies treated with physiotherapy in PPC; (2) describe the physiotherapy interventions applied to these patients and the effects achieved in the child and his/her family after rehabilitation; and (3) identify the knowledge of physiotherapists on PPC.

## 2. Materials and Methods

### 2.1. Search Strategy

This systematic review was conducted through a literature search between February and April 2021. This search was performed in different online scientific databases related to Health Sciences: Medline/PubMed, Cochrane Library, PEDro, CINAHL, and Scopus. The search strategy and the key terms used were adapted to the different databases (Table 2). The descriptors contained in each column were combined with each other using the Boolean operator “OR”. The group of descriptors of each category was crossed with the other groups using the Boolean operator “AND”.

### 2.2. Inclusion and Exclusion Criteria

Each article was selected and analyzed for inclusion by two independent authors in parallel. This systematic review included all those articles that met the following inclusion criteria:

- Qualitative, quantitative, and mixed-methods studies.
- Study population exclusively composed of children, children, and their families or physiotherapists.
- The sample must either have some disease susceptible to palliative care or be part of a palliative care program.
- Articles in which there is some relationship between physiotherapy and PPC.
- Articles published in the last 10 years.
- Articles published in English or Spanish.
- Articles available in full text.

On the other hand, the present study excluded reviews, studies that only addressed medical or pharmacological interventions, and publications that did not meet the inclusion criteria. This review is reported according to Preferred Reporting Items for Systematic Reviews and Meta-Analyses (PRISMA) guidelines.

## 3. Results

### 3.1. Article Selection Process

#### A Total of 622 Studies Were Initially Identified after the Search

Search through Medline/PubMed: The filter of articles published in the last 10 years was directly applied. A total of 275 results were obtained after combining the different key terms in the search. After reading the titles, 198 articles were discarded, and another 62 were discarded after reading the abstracts. Of the 15 remaining articles, the full text was read, discarding 11 of them for not meeting the inclusion criteria (seven for adult population and four for being systematic reviews). Finally, four articles found in PubMed were included.

Search through Cochrane Library: Firstly, a simple search was conducted, finding no results. With the advanced search, eight results were obtained, which were all discarded after reading the titles.

Search through PEDro: Following the same procedure, this database did not produce any suitable article, neither through the simple search nor through the advanced search. Only one result was obtained, although it was a list of guidelines; thus, it was discarded.

Search through CINAHL: The filter for the key terms to appear in the title was applied. A total of 44 results were obtained, of which 5 were discarded for being repeated, 34 after reading the title, and 4 after reading the abstract; finally, 1 article was read in full text and was included in the review.

Search through Scopus: A total of 294 results were obtained, of which 117 articles were discarded for being repeated. After reading the titles, 110 were discarded, and another 54 were discarded after reading the abstracts. Of the 13 remaining articles, which were read in full text, 11 were discarded for not meeting the inclusion criteria (eight for including only pharmacological measures for symptom control and three for being systematic reviews). Finally, two articles found in Scopus were included in the present systematic review.

**Figure 1:**
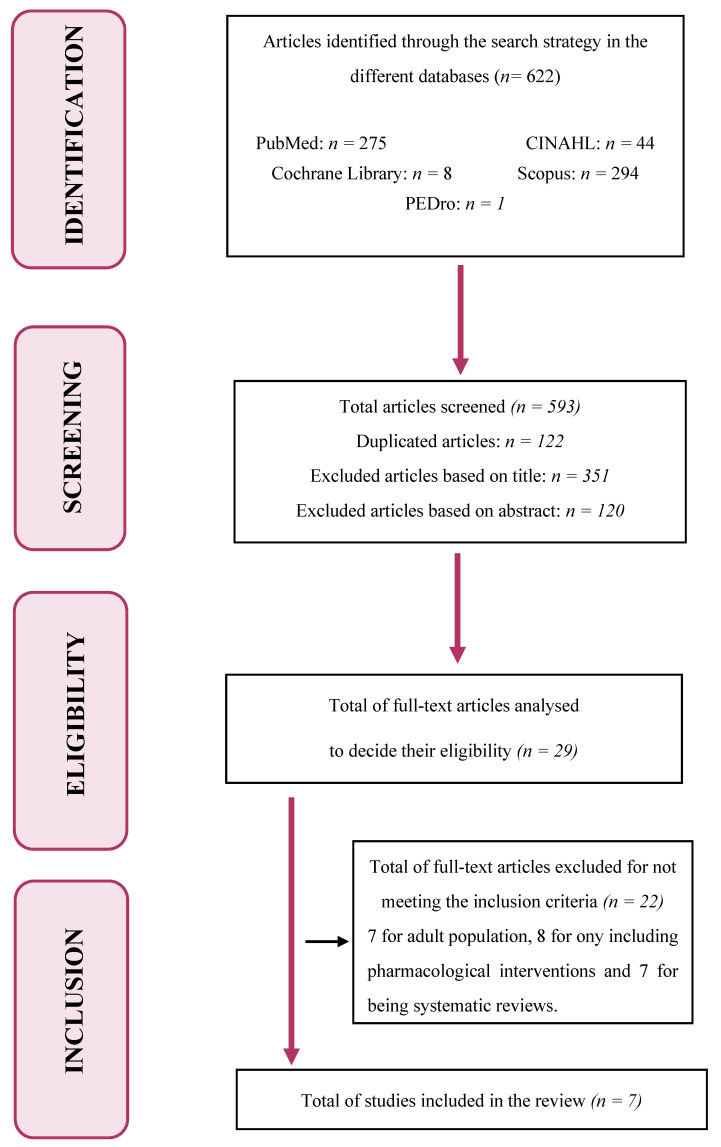
Flowchart of the article selection process.

After the article selection process and the application of the inclusion and exclusion criteria, a total of seven studies were finally included in the review (Figure 1). Of these seven studies, five were quantitative (three descriptive and two quasi-experimental or pre-post), one was qualitative, and one was a clinical case.

The methodological quality of the selected articles was determined using the guidelines of the Consolidated Criteria for Reporting Qualitative Research (COREQ) for the qualitative studies, Strengthening the Reporting of Observational Studies in Epidemiology (STROBE) for the descriptive quantitative studies, Consensus-based Clinical Case Reporting (CARE) for the clinical cases, and Transparent Reporting of Evaluations with Nonrandomized Designs (TREND) for the quasi-experimental/pre–post studies. These scales measure the validity and interpretability of the results of the studies through a series of items (specifically 32, 22, 13, and 22 items, respectively). Table 3 shows the scale used for each article, as well as the score obtained after their evaluation.

The main characteristics of the selected studies (population, main intervention, data gathering, and results) are presented in Table 4.

### 3.2. Study Population

A total of 202 individuals were included, of whom 144 were children, 14 were parents, and 44 were physiotherapists. The sample size of the studies was not homogeneous, ranging between 1 [19] and 89 [22] participants. The age of the children was also very variable in the different studies, ranging between 0.6 months [20] and 17 years [17], that of the parents was between 29 and 51 years [18], and that of the physiotherapists was between 20 and 50 years [21]. The described data are presented in Table 5.

### 3.3. Study Variables and Data Gathering

The main variables analyzed in the studies were the experience and perspectives of the parents about the home physical rehabilitation, the management of the different symptoms of the children in PPC from physiotherapy, and the knowledge of the physiotherapists about PPC.

The experience and perspective of the parents about the home physical rehabilitation were evaluated in two of the articles [16,18] included in this review. The article of Dangel et al. [16] gathered the data through questionnaires filled by the parents, whereas in the study of Rico et al. [18], the researchers carried out interviews with the parents to collect the information.

Four studies [17,19,20,22] evaluated the management of the different symptoms of the children in PPC from physiotherapy. In the study of Genik et al. [17], the *Pediatric* Quality of Life Inventory Cancer Module assessed the quality of life of the child and of the parent, the PainSquad and Faces Pain Scale evaluated pain and the impact on the daily life of the child, and the Children’s Fear Scale assessed the concern and fear of the children. The study of Weingarten et al. [19] evaluated pain and the quality of life, and the study of Hully et al. [20] assessed pain and respiratory, nutritional, and motor management; both studies used questionnaires that were filled by the study girl and parents, respectively. To evaluate the motor functions and spasticity in the study of Sarmad et al. [22], the authors used the Gross Motor Function Classification System and the Modified Tardieu Scale.

The knowledge of the physiotherapists about PPC was only evaluated in one study [21] through socio-professional questionnaires and the Bonn Palliative Care Knowledge Test.

### 3.4. Pathologies Treated with Physiotherapy in Pediatric Palliative Care (PPC)

Cerebral palsy (CP) and cancer are the most studied pathologies. Children with CP are included in two studies [18,22], although in a general manner, without specifying the type of paralysis. The study of Sarmad et al. [22] indicates that the children have a level IV and V GMFCS, which are the most severe levels regarding the motor functions. Cancer is addressed in another two studies [17,19], with leukemia being the type of cancer tackled in both studies. Moreover, the study of Genik et al. [17] includes cases of lymphoma, carcinoma, and sarcoma.

Other pathologies treated with physiotherapy in PPC are SMA1 [20], NBIA [16], severe stroke, polymalformative syndrome, Patau’s syndrome, hydranencephaly, non-identified mitochondrial disorder, and Tay-Sachs disease [18].

### 3.5. Physiotherapeutic Interventions in PPC

Two of the studies [20,22] introduced techniques of neurological physiotherapy in their interventions. None of them used neurological physiotherapy as an exclusive treatment. Hully et al. [20] included respiratory physiotherapy, whereas Sarmad et al. [22] included family training. Both studies highlight a correct postural management for spasticity and to prevent possible deformities of the child; only Sarmad et al. [22] underline techniques aimed at neurodevelopment and sensory stimulation. None of the studies mention the protocol or the duration of the intervention.

As was previously mentioned, respiratory physiotherapy was used with neurological physiotherapy in the study of Hully et al., where 91% of the patients received it (90% at home, and 80% between 3 and 7 days per week); each session lasted about 10 min, and the specific techniques used in each session are not specified [20]. Although the study of Dangel et al. [16] does not mention any physiotherapeutic intervention, it does refer to the physiotherapist as an important part of the multidisciplinary team, and the discussion points out respiratory physiotherapy as one of the most important therapies for their children aimed at preventing and treating atelectasis.

Therapeutic massage is the main intervention in the study of Genik et al. [17] for the management of pain and the quality of life of the child. It consisted in one hour of therapeutic massage per week, either in the hospital or at home depending on the needs of each child, for one month, with a follow-up of 4–6 weeks. Neither the localization nor the protocol of the massage technique are specified. Moreover, the therapeutic massage is combined with neurological and respiratory physiotherapy in the study of Hully et al. [20] for the management of constipation and the intestinal function, although the protocol is not indicated.

Weingarten et al. [19] aimed to develop a VR program in PPC, and this is the first clinical case to verify whether VR is a useful therapeutic tool for symptom control and for the improvement of the quality of life. The experience of virtual reality was guided by a young company called Wishplay in the hospital room.

### 3.6. Effects Observed in the Children in Palliative Care and their Families after the Physiotherapeutic Intervention

Neurological physiotherapy had significant positive effects in the two studies that used it [20,22]. In the study of Hully et al. [20], over 80% of the parents considered that motor physiotherapy provided well-being to their children and over 70% stated that it was not painful. In the study of Sarmad et al. [22], 100% of the children presented significant changes in the gross motor activities, and spasticity decreased in 77.53% of the patients.

Over 80% of the parents considered that respiratory physiotherapy helped their children with the mucociliary clearance in the airways, although they also found it tiring [20]. Over 60% of the parents felt comfort and did not consider it painful. The study of Dangel et al. [16] highlighted respiratory rehabilitation as the second most important form of therapy to prevent the early death of the patient.

The participants of the study of Genik et al. [17] reported a significant decrease of pain after two sessions of massage therapy, as well as a reduction of the children’s concern after only one session. However, the effects did not prevail in the long term, since there were no significant changes between the beginning and follow-up of the study, or in the quality of life. In the study of Hully et al. [20], 16 out of 22 parents considered constipation as uncomfortable for their children, who obtained a significant improvement with the abdominal massage.

In the study of Weingarten et al. [19], virtual reality distracted the patient from her usual environment, which was a hospital room. Before the sessions, she felt nausea and headaches and was lonely most of the time. During the sessions, she got distracted from the pain and loneliness that she was feeling. Moreover, she preferred being alone rather than accompanied by a professional during the VR session.

With respect to the experience and perspectives of the families, the study that best describes them is that of Rico et al. [18], although the study of Dangel et al. [16] also mentions some of them. For the parents, physical rehabilitation is a “technique” that improves the quality of life of the child, is part of his/her life, and allows him/her to connect with the environment. Many parents stated that finding the best physical therapy for their child was not easy, and that the experiences had not always been good. However, they also pointed out that physical rehabilitation improved the motor skills of the child and, in the end, became a daily requirement and obligation [18]. Furthermore, the physiotherapists taught the techniques to the parents, involving them in the treatment of their children, making them feel more supported, and improving their capacity to solve any incident. All the parents highlighted the open and positive attitude of the professionals [16,18]. The parents perceived the help provided by the PPC unit as an essential and necessary source of support. For the parents, physical therapy at home integrated health care in the environment of the child, helping to maintain their bonds with the family, reducing the expenditures, and preserving the capacity of the child in his/her own environment until the last moment [18].

### 3.7. Knowledge of the Physiotherapists about PPC

The article of Oliveira et al. [21] is the only one that evaluated the knowledge of the physiotherapists who work in PPC, through the application of a questionnaire. A total of 93.2% of the physiotherapists had not received training in PPC during the university degree, whereas 34.1% of the respondents had received it after graduating. A total of 90.5% thought that non-pharmacological therapies such as physiotherapy were important for pain management. Another result worth highlighting is that 100% of the physiotherapists believed that communication skills were very important and could be learned.

## 4. Discussion

The present study allowed evaluating the available scientific evidence on physiotherapy in children who require palliative care. As was previously mentioned, PPC is a set of strategies that are applied to those children with a severe or life-threatening disease with the aim of controlling the symptoms and improving the quality of life of both the child and of his/her family. They can be initiated at any age and at any stage of the disease; they can even be provided along with a curative treatment. The strategies used for symptom control include physical rehabilitation, which can be carried out by physiotherapists.

To delve into this aspect, the literature of the last 10 years was reviewed, observing that only seven articles met the inclusion criteria. Most of the publications were discarded based on the fact that the main treatment carried out in the children was pharmacological or the study population was constituted by adults. Moreover, many articles extrapolated the interventions conducted in the adult population to the children population, without considering that children and adolescents need to adapt the strategies followed in palliative care to their physical, emotional, and cognitive development.

There are different ways of analyzing and understanding the role of physiotherapy in PPC: from studies conducted in children through the experiences of the families of the patients and even directly from the professionals who work in the PPC units. This is why the population of the present review is so variable and heterogeneous. In children, the age range varies between 0.6 months [20] and 17 years [17], that of the parents with children in PPC varies between 29 and 51 years [18] and that of the physiotherapists who work in PPC varies between 20 and 50 years [21]. Regarding sex, conclusive information could not be obtained, since some studies did not include these data; however, there was a predominance of females in the different populations analyzed.

Cerebral palsy (CP) and cancer are the most studied pathologies [17,22], followed by SMA1 [20]. Despite the fact that cancer is in the first line, CP is abundant in more cases, which shows that researchers also work on disabling and life-threatening pathologies, and only on cancer. However, there are multiple pathologies susceptible to require PPC that were not included in this revision.

Symptom control was the most studied variable [17,19,20,22]. Pain and quality of life also stand out, which were evaluated with different methods. Genik et al. [17] used PainSquad, FPS-R, and the PedsQL Cancer Module, Hully et al. [20] employed questionnaires filled by the parents, and Weingarten et al. [19] applied questionnaires completed by the study assistant. The management of the motor functions was analyzed by Sarmad et al. [22] with GMFCS and MTS, whereas Hully et al. [20] explored this and respiratory management with questionnaires filled by the parents.

Despite the variability in the analyzed pathologies, many symptoms in children in PPC are common, such as pain [17,20], respiratory symptoms [16,20], neurological symptoms (spasticity) [20,22], and psychological symptoms (concern and fear) [17], although they must be addressed individually in each child and disease.

Respiratory physiotherapy and the techniques aimed at controlling neurological symptoms are the most prevalent specialties of physiotherapy in PPC. Neurological physiotherapy had significant positive effects in the two studies that used it (*p* < 0.05) [20,22]. Both studies included postural management for spasticity and to prevent possible deformities of the child. It is worth highlighting the techniques aimed at neurodevelopment and sensory stimulation used in the study of Sarmad et al. [22]. Respiratory physiotherapy also had significant benefits for mucociliary clearance in the airways [20] and for the prevention and treatment of atelectasis [16]. The parameters of the interventions are not specified.

Several of the studies included in this review highlight the relevance of family training [16,18,20,22], stating that the education and training of the parents for the application of motor and respiratory techniques is very important, since they are the main caregivers of the child. The management of secretions, postural management, control of spasticity or muscle tone, and ensuring that the child is well positioned to rest or eat and that he/she develops as few deformations as possible are some of the aspects that physiotherapists teach the families.

Another study variable was the experiences and perspectives of the families about physiotherapy in the palliative care of their children. Rico et al. [18] and Dangel et al. [16] agreed that physical rehabilitation improved the motor skills of the child and, therefore, became a daily requirement and obligation. The fact that this variable had been included highlights that, in PPC, the care unit is the child and his/her family.

Despite not being among the study objectives, another key result for PPC was found, which is the fact that the treatments were carried out at home and in the environment of the child [16,17,18,20,22]. For the parents, physical therapy at home integrated health care in the environment of the child, helping to maintain bonds with his/her family, reducing expenditures, and maintaining the capacity of the child in his/her own environment until the last moment [18].

Another aspect worth pointing out is that physiotherapy in PPC can have different objectives, such as curing or alleviating, accompanying and maintaining the child. This becomes evident in this review. For example, in the study of Hully et al. [20], some children with SMA1 die even before the end of the study, as well as in the study of Dangel et al. [16]. Other studies, such as that of Sarmad et al. [22], included severe chronic pathologies such as CCP, which is equally susceptible to receive palliative care, although the objectives were different. To sum up, the professional attitudes of physiotherapists must change toward the final care of life and continuously adapt to the needs of the children.

The last study variable was the knowledge of the physiotherapists about PPC. The article of Oliveira et al. [21] is the only one that evaluates it, showing that 93.2% of the participants had not received training in PPC during the university degree, whereas 34.1% had received it after graduating.

### Limitations of the Study and Future Research Lines

One of the main limitations is that very few studies met the established inclusion criteria. Therefore, among others, a clinical case was included, which, despite its low methodological quality, was considered to provide relevant information related to the study topic. Secondly, none of the included studies had a control group, although it is important to take into account that such studies do not exist due to ethical conflict, since no child under palliative care can be left without treatment.

Another limitation was that children under palliative care require a multidisciplinary approach, and thus, it was not possible to carry out the physiotherapeutic intervention alone in most of the studies; therefore, it is not possible to know whether the obtained results are exclusively due to our treatment.

The population sample in several studies was very small and not enough to extrapolate the results to the general children population.

The present review suggests that there is still much to work on to determine the role of physiotherapy in PPC. In order for this to be attained, certain barriers that hinder research in this topic must be overcome, such as the disapproval of trials by the ethics committees, the absence of funding or other types of resources, and the lack of potential participants. Further research is needed to obtain standardized protocols for the realization of physiotherapy programs that improve the quality of life and reduce the symptoms in children with a disease susceptible to receive palliative care. Thus, it would be relevant to carry out a clinical trial to give more attention to these patients and to study in depth the benefits that physiotherapy can have within its therapeutic approach.

## 5. Conclusions

The findings of this review indicate the lack of an adequate evidence foundation for physiotherapy in PPC. There are very few research projects and training programs for physiotherapists in this specific field, which implies that it is underused in children under palliative care and/or at the end of life, despite the reported benefits.

## Figures and Tables

**Table 1:** Classification of the ACT and RCPCH regarding patients susceptible to receive PPC [5].

Groups	Definition	Examples
Group 1	Potentially deadly diseases for which curative treatment may be feasible but may also fail. During the curative treatment, palliative care may be required during an acute crisis or if the treatment fails.	Cancer Heart, liver. or kidney failure Infections
Group 2	Diseases that require long periods of intensive treatment aimed at prolonging life and enabling participation in normal activities but in which early death is possible.	Cystic fibrosis Extreme prematurity Cardiovascular anomalies
Group 3	Progressive diseases with no options of curative treatment, where treatment is exclusively palliative since diagnosis and may extend for many years.	Neuromuscular or neurodegenerative disorders Progressive metabolic disorders Chromosomal anomalies
Group 4	Irreversible non-progressive diseases that cause a severe disability, which leads to an extreme vulnerability to suffer from health complications and to the probability of early death.	Severe cerebral palsy Congenital malformations Brain or spinal cord lesions

**Table 2:** Search strategy of studies on the role of physiotherapy in PPC.

Pediatric Descriptors	Descriptors of Palliative Care and Susceptible Diseases	Descriptors of Symptom Control	Descriptors of Physiotherapy
Pediatric Child Children Infant Childhood	Palliative care Terminal conditions Oncology/cancer Cerebral palsy Neuromuscular or neurodegenerative disorders Extremely premature Congenital malformations	Symptom management Symptom control Physical symptoms	Physical therapy Physiotherapy Exercise therapy Rehabilitation

**Table 3:** Evaluation of the methodological quality with STROBE for the descriptive quantitative studies, CARE for the clinical cases, TREND for the quasi-experimental studies, and COREQ for the qualitative studies.

Year and Authors	Study Design	Scale	Score
Dangel et al., 2019	Descriptive quantitative study	STROBE	13/22
Genik et al., 2019	Quasi-experimental study	TREND	16/22
Rico et al., 2019	Qualitative study	COREQ	26/32
Weingarten et al., 2019	Clinical case	CARE	9/13
Hully et al., 2020	Descriptive quantitative study	STROBE	18/22
Oliveira et al., 2021	Descriptive quantitative study	STROBE	17/22
Sarmad et al., 2021	Quasi-experimental study	TREND	16/22

**Table 4:** Main characteristics of the selected articles.

Year and Authors	Study Design	Population	Main Intervention	Study Variables	Data Gathering	Results
Dangel et al., 2019 [16]	Descriptive quantitative study	9 children aged 7–14 years with neurodegeneration with brain iron accumulation (NBIA)	— Any main intervention applied	Home pediatric palliative care for children with NBIA and their families	Questionnaires filled by parents. The study was conducted between 1998 and 2018	PPC reduces pain and improves symptom control in most cases, but not in all The quality of the home pediatric palliative care provided by the multidisciplinary team was valued as high, with very satisfied parents Regarding the rehabilitation, respiratory physiotherapy is highlighted as one of the most important therapies to prevent the early death of the patient
Genik et al., 2019 [17]	Pre–post pilot study of a single group	8 children with cancer: *n* = 1 carcinoma, *n* = 4 leukemia, *n* = 2 lymphoma, *n* = 1 sarcoma, (aged 10–17 years) who had been referred to PPC and one of the parents	Therapeutic massage (1 h) once per week in the hospital or at home for one month. Follow-up of 4–6 weeks	Quality of life of the child and his/her parents Pain and impact on the daily life of the child Concern and fear of the children	PedsQL Cancer Module PainSquad and FPS-R CFS	Significant decrease of the children’s pain and concern (*p* = 0.03) The effects do not persist in the long term No significant changes were observed in the quality of life
Rico et al., 2019 [18]	Qualitative study	14 parents and 11 children in PPC (*n* = 5 CCP, *n* = 1 severe stroke, *n* = 1 polymarformative syndrome, *n* = 1 Patau’s syndrome, *n* = 1 hydranencephaly, *n* = 1 non-identified mitochondrial disorder, *n* = 1 Tay-Sachs disease)	Any main intervention applied	Experience and perspective of the parents about the home physical rehabilitation in PPC	Non-structured and semi-structured interviews for 8 months	The physical rehabilitation increased the quality of life of the children and improved their motor skills The home physical rehabilitation facilitated the learning of the techniques by the parents in the usual environment of the child In the socio-family environment, the home physical rehabilitation helped to maintain bonds with the family, reduced expenditures, and helped the child to better adapt to the environment
Weingarten et al., 2019 [19]	Clinical case	12-year-old girl with acute myeloid leukemia who participates in a PPC program	Virtual reality in the hospital room	Symptom control Quality of life	Questionnaire before and after the VR experience	VR favored distraction and made her believe she came out of the room Pain decreased during VR Better experience with VR alone and unaccompanied by a professional.
Hully et al., 2020 [20]	Multicenter prospective cohort study	37 SMA-1 patients (20 girls and 17 boys) with an average age of 3 months	Respiratory physiotherapy from 3 per week to every day, 10 min approx. Motor physiotherapy (postural management). Abdominal massage	Respiratory, nutritional, and motor management Child pain and comfort	Questionnaires filled out by parents and notes from professionals 2012–2016	*p* < 0.05 - Airway clearance, comfort and no pain with respiratory physiotherapy, but tiring - Well-being, no pain with physical motor - Modifying feeding and massaging abdomen improve bowel function and constipation
Oliveira et al., 2021 [21]	Descriptive quantitative study	44 physiotherapists (35 women and 9 men) between 20 and 50 years old from the pediatric and NICU units	—	Knowledge of physiotherapists about PPC	Socio-professional questionnaires BPW test	93.2% have not received training on PPC during the degree, 34.1% have received it after graduating, 90.5% considered non-pharmacological therapies such as physical therapy important for pain management
Sarmad et al., 2021 [22]	Quasi-experimental study	89 children aged 1–3 years with spastic cerebral palsy (level IV or V GMFCS)	Neurodevelopment and sensory stimulation Family training on postural management in different activities Duration: 3–4 h per week, for 3 months	Management of the mothers on the motor functions and spasticity in the time when their children are not in physiotherapy	GMFM before and after the intervention of the physiotherapist MTS	Adequate management of the child by the mothers led to significant changes in the gross motor activities in 100% of the children (*p* = 0.00), whereas the spasticity decreased in 77.53% of the patients.

NBIA: neurodegeneration with brain iron accumulation; PPC: pediatric palliative care; PedsQL: Pediatric Quality of Life Inventory; FPS-R: Faces Pain Scale; CFS: Children’s Fear Scale; CCP: children cerebral palsy; VR: virtual reality; GMFCS: Gross Motor Function Classification System; GMFM: Gross Motor Function Measure; MTS: Modified Tardieu Scale.

**Table 5:** Characteristics of the study population.

Population Type	Number of Participants	Age	Sex
Children	144	0.6 months–17 years	M: 22 *
			F: 24 *
Parents	14	21–59 years	M: 4
			F: 10
Physiotherapists	44	20–50 years	M: 9
			F: 35
Total	202		M: 35 *
			F: 69 *

M: male; F: female; * Information about the sex of the participants of the studies of Dangel et al. [16] and Sarmad et al. [22] is not available.
